# Long-term PTSD prevalence and associated adverse psychological, functional, and economic outcomes: a 12–15 year follow-up of adults with suspected serious injury

**DOI:** 10.1080/20008066.2024.2401285

**Published:** 2024-09-19

**Authors:** Jeanet F. Karchoud, Juanita Haagsma, Irina Karaban, Chris Hoeboer, Rens van de Schoot, Miranda Olff, Mirjam van Zuiden

**Affiliations:** a Psychiatry, Amsterdam Public Health, Amsterdam UMC, University of Amsterdam, Amsterdam, The Netherlands; b Department of Public Health, Erasmus MC, University Medical Center Rotterdam, Rotterdam, The Netherlands; c Department of Methods and Statistics, Utrecht University, Utrecht, The Netherlands; d ARQ National Psychotrauma Centre, Diemen, The Netherlands; e Department of Clinical Psychology, Utrecht University, Utrecht, The Netherlands

**Keywords:** PTSD, trauma, anxiety, depression, alcohol use, well-being, quality of life, economic costs, longitudinal, gender, TEPT, trauma, ansiedad, depresión, consumo de alcohol, bienestar, calidad de vida, costos económicos, longitudinal, género

## Abstract

**Background:** An increasing number of longitudinal studies investigates long-term PTSD, related outcomes and potential gender differences herein. However, a knowledge gap exists when it comes to studies following individual civilian trauma beyond a decade post-trauma.

**Objective:** To investigate the long-term PTSD prevalence, associated adverse psychological, functional and economic outcomes related to (suspected) serious injury of 12–15 years ago in Dutch adults, as well as potential gender differences herein.

**Method:** *N =* 194 trauma-exposed adults (34% women) admitted to an emergency department following suspected serious injury completed a follow-up assessment 12–15 years (*M *= 14.30, *SD* = 1.00) post-trauma. Participants completed assessments of clinician-rated PTSD symptom severity, as well as self-report questionnaires on psychological, functional and economic outcomes.

**Results:** Nine participants (4.8%) fulfilled the DSM-5 diagnostic criteria for PTSD related to the index trauma of 12–15 years ago. Results showed that PTSD symptom severity (CAPS-5) was significantly associated with more severe symptoms of anxiety (HADS) and depression (QIDS), lower well-being (WHO-5) and (health-related) quality of life (WHOQOL; EQ-5D-5L), but not with alcohol use (AUDIT), productivity loss at work (iPCQ) and health care use (iMCQ). No significant gender differences in the long-term PTSD prevalence nor in its related psychological, functional and economic outcomes were found.

**Conclusions:** Our findings underscore the long-term presence of PTSD and associated adverse psychological and functional outcomes in a proportion of adults who experienced (suspected) serious injury over a decade ago. PTSD is already widely recognized for its substantial impact in the aftermath of a trauma. The current study emphasizes the potential long-term consequences of individual civilian trauma, highlighting the importance of accurate screening and prevention for PTSD.

## HIGHLIGHTS

- We investigated long-term PTSD and associated adverse outcomes 12–15 years post-trauma.
- 4.8% had PTSD 12–15 years following suspected serious injury.
- Higher PTSD symptoms were associated with adverse psychological and functional outcomes.

## Abstract (es)

**Antecedentes:** Un número cada vez mayor de estudios longitudinales investigan el TEPT a largo plazo, resultados relacionados y las posibles diferencias de género en este sentido. Sin embargo, existe una brecha de conocimiento cuando se trata de estudios posteriores a traumas civiles individuales más allá de una década después del trauma.

**Objetivo:** Investigar la prevalencia del TEPT a largo plazo, los resultados psicológicos, funcionales y económicos adversos asociados relacionados con lesiones graves (presuntas lesiones) de hace 12 a 15 años en adultos holandeses, así como las posibles diferencias de género en este sentido.

**Método:** Adultos expuestos a traumas, *N* = 194, (34% mujeres) ingresados ⁣⁣en un departamento de emergencias después de una presunta lesión grave completaron una evaluación de seguimiento 12 a 15 (*M* = 14,30, *SD* = 1,00) años después del trauma. Los participantes completaron cuestionarios para evaluar la gravedad de los síntomas de TEPT, así como cuestionarios de auto-reporte sobre los resultados psicológicos, funcionales y económicos.

**Resultados:** Nueve participantes (4,8%) cumplieron los criterios diagnósticos del DSM-5 para el TEPT relacionado con el trauma índice de hace 12–15 años. Los resultados mostraron que la gravedad de los síntomas de TEPT (CAPS-5) se asoció significativamente con síntomas más graves de ansiedad (HADS) y depresión (QIDS), menor bienestar (WHO-5) y calidad de vida (relacionada con la salud) (WHOQOL; EQ-5D-5L), pero no con el consumo de alcohol (AUDIT), la pérdida de productividad en el trabajo (iPCQ) y el uso de atención médica (iMCQ). No se encontraron diferencias significativas de género en la prevalencia de TEPT a largo plazo ni en sus resultados psicológicos, funcionales y económicos relacionados.

**Conclusiones:** Nuestros hallazgos subrayan la presencia a largo plazo de TEPT y los resultados psicológicos y funcionales adversos asociados en una proporción de adultos que experimentaron (presuntas) lesiones graves hace más de una década. El TEPT ya es ampliamente reconocido por su impacto sustancial como consecuencia de un trauma. Este estudio enfatiza las posibles consecuencias a largo plazo del trauma individual en civiles, destacando la importancia de la precisión en la detección y prevención del TEPT.

Posttraumatic stress disorder (PTSD) is a psychiatric disorder that can develop in individuals who experience a potentially traumatic event (PTE). Globally, 70% of adults experience at least one PTE in their lives (Kessler et al., 2017), with an estimated lifetime prevalence of 80% in the Netherlands (de Vries & Olff, 2009). The conditional risk for developing PTSD after a PTE differs between types of events with an average of 4% (Liu et al., 2017). PTSD is characterized by recurrent involuntary memories in the forms of intrusions, nightmares or flashbacks, alongside avoidance of stimuli associated with the trauma, alterations in mood and cognition, and heightened arousal and reactivity (American Psychological Association [APA], 2013). Once established, PTSD often follows a chronic course, with symptoms persisting for years to decades following the traumatic event (Kessler et al., 2017). There is a growing number of longitudinal studies providing insights into the prevalence of such long-term PTSD, particularly in the context of uniformed services and collective trauma. For instance, one study observed a PTSD prevalence of 4.5% in male US veterans and 6.1% in female US veterans 40 years after the Vietnam war (Marmar et al., 2015). Another study reported a PTSD prevalence of 21% in Italian survivors 36 years after a large-scale tsunami (Favaro et al., 2004). However, there is a noticeable lack of studies examining long-term PTSD prevalence in civilian populations experiencing individual trauma, such as traffic accidents or physical assault. The final assessments of longitudinal studies in these populations usually occurred within a year (Heron-Delaney et al., 2013). The longest follow-up period in the current literature is 8 years after physical assault in Norwegian civilians, where a PTSD prevalence rate of 19% was reported (Johansen et al., 2013). Thus, data on the long-term prevalence of PTSD beyond 10 years after individual civilian trauma is currently lacking.

Research shows that long-term PTSD is associated with a wide variety of adverse outcomes regarding the individual's emotional well-being, and daily functioning, as demonstrated by multiple long-term studies conducted in occupational and collective trauma samples between 14 and 40 years post-trauma (Adams et al., 2019; Holtslag et al., 2007; Johansen et al., 2013; Kellezi et al., 2017; Marmar et al., 2015; Munyandamutsa et al., 2012; Nickerson et al., 2014; Soberg et al., 2015; Vles et al., 2005). This includes for example high comorbidity of psychological disorders such as anxiety and depression (Adams et al., 2019; Marmar et al., 2015; Munyandamutsa et al., 2012). This higher comorbidity of depression and anxiety was also found 8 years following individual civilian trauma (Johansen et al., 2013). Another study found that more severe PTSD symptoms were related to more alcohol use 2 years following traumatic injury (Nickerson et al., 2014). Nonetheless, although likely, it remains uninvestigated whether observed associations between long-term PTSD and these related psychological outcomes also persist beyond a decade after individual civilian trauma. The same applies to health-related quality of life (Geraerds et al., 2020; Soberg et al., 2015) and other quality-of-life domains. Furthermore, information on the relationship between long-term PTSD symptoms and economic outcomes is limited. Given that research reported higher overall health care costs in individuals with PTSD, driven by increased use of both mental health and non-mental health care (Bothe et al., 2020; Harper et al., 2022), it is worth investigating whether this also applies to long-term PTSD.

A growing number of studies examines the influence of sex and gender to comprehend individual variability in prevalence, manifestation and aetiology of PTSD (Haering et al., 2022). This is particularly relevant as there is meta-analytic evidence for a higher conditional PTSD risk in women compared to men (Tolin & Foa, 2008). However, a recent systematic review and meta-analysis on longitudinal studies with multiple assessments in trauma-exposed individuals showed that this increased PTSD prevalence in women might not persist long-term (Haering et al., 2022). In this meta-analysis, no differences in PTSD prevalence between men and women were observed in the few injury samples assessing PTSD at 2 and 5 years post-trauma, whilst higher PTSD prevalence was present in women at 1–6 months and 1–2 years post-trauma (Haering et al., 2022). On the other hand, the meta-analysis showed that women had consistently higher PTSD symptom severity until 2–5 years post-trauma (Haering et al., 2022). Overall, there is still limited knowledge on the long-term differences in PTSD prevalence between men and women. Moreover, it has only been scarcely investigated whether men and women differ in long-term PTSD-related adverse psychological, functional and economic outcomes. There are studies reporting gender differences in psychological comorbidity in people with PTSD (i.e. Brady et al., 2000; Kessler et al., 2013 higher in men; i.e. Spinhoven et al., 2014 higher in women), however, it remains uninvestigated whether this applies to the long-term as well.

The goal of the current longitudinal follow-up study was to investigate the long-term prevalence of PTSD related to a traumatic event resulting in (suspected) serious injury of 12–15 years ago. Moreover, we examined cross-sectional associations between long-term PTSD symptom severity related to the traumatic event of 12–15 years ago and psychological (anxiety, depression, alcohol use), functional (well-being, general and health-related quality of life) and economic (productivity loss and health care use) outcomes. We also examined whether gender differences in long-term PTSD prevalence, and associations between long-term PTSD symptom severity and the investigated psychological, functional and economic outcomes occurred.

## 1. Methods

### 1.1. Participants and study design

The current study included *N = *194 trauma-exposed adults (34% women, mean age at follow-up = 54 years) admitted to the emergency department (ED) following (suspected) serious injury who completed a follow-up assessment at 14.3 years (*SD* = 1.00, range 12–15) post-trauma. Participants were part of the TraumaTIPS cohort, a large prospective cohort study on psychopathology following suspected serious injury conducted between 2005 and 2009 (‘The Incidence, Prediction and Prevention of Post-trauma Psychopathology Study’; Mouthaan et al., 2014). The original cohort consisted of *N = *852 participants who were included after the presentation by ambulance or helicopter for acute emergency medical care to one of two level-1 trauma centres in Amsterdam, the Netherlands (hospitals Academic Medical Center and VU University Medical Center). Inclusion criteria of the TraumaTIPS cohort were: age 18 years or older; (suspected) injuries sustained in a traumatic event according to DSM-IV PTSD A1 criterion (Weathers et al., 2001); sufficient understanding of Dutch language. Exclusion criteria were: current severe psychiatric symptoms (a psychotic or bipolar disorder, depressive disorder with psychotic features, evidence of intentional self-inflicted injury); moderate-severe traumatic brain injury; permanent residency outside the Netherlands. Participants were additionally excluded from the follow-up study in case of suspected severe neurological conditions clearly impairing cognition (e.g. dementia). The TraumaTIPS cohort study was approved by the Medical Ethics Review Committee of the Academic Medical Centre and VU University Medical Center hospitals. The current follow-up study was exempted from formal ethical review by the Medical Ethical Review Committee of the Amsterdam University Medical Centers, location Academic Medical Centre, upon merging of the two aforementioned hospitals (W20_035#20.063).

### 1.2. Procedures

Participants were included within the current follow-up study between 2021 and 2022. We included all reachable, eligible, and willing participants of the TraumaTIPS cohort study (Mouthaan et al., 2014). We approached *n = *543 participants (63.7%) of the cohort who previously provided permission to be approached for follow-up measurements in their informed consent and who had not formally withdrawn from participation during the original study. They were contacted via telephone and mail using their prior contact information. We verified their identity via telephone using personal details and by probing about their past traumatic event in relation to the cohort participation (i.e. the index trauma). Information about their index trauma and their country of origin was retrieved from the original TraumaTIPS cohort data. There were *n = *171 (31.5%) participants that could not be reached via prior contact information. Furthermore, upon establishing contact *n = *12 (2.2%) former participants were excluded due to impaired cognition or language and *n = *27 (4.5%) former participants due to other reasons including death. Additionally, *N = *136 (25%) former participants declined to participate in the current study. Upon successfully confirming participants’ identity and eligibility, study information about the TraumaTIPS Follow-up study was sent via email, including a personalized link to provide written informed consent via a secure online platform (Castor Electronic Data Capture [EDC], 2019). This resulted in *n = *194 (36.3%) participants who provided informed consent. After providing informed consent, participants received a personal link providing access to online self-report questionnaires on several psychological, functional, economic and sociodemographic outcomes including gender via Castor EDC. Moreover, an online meeting for the clinical interview was scheduled to assess whether participants met the diagnostic criteria for PTSD, their PTSD symptom severity and potentially additional trauma exposure after their initial index trauma related to the cohort participation. Participants provided permission to audiotape the interviews to assess interrater reliability. All assessments were in Dutch. Participants were asked to fill out the questionnaires before the clinical interview or shortly after. The online questionnaires had a forced entry, meaning that missing data only occurred when participants stopped filling out the questionnaires. *N = *9 (6.2%) participants were excluded from analyses because they did not complete the interview and questionnaires. In total, *n = *185 (93.9%) participants who completed the CAPS-5 interview were included within our analyses. Among these participants, there was *n *= 1 participant who did not complete the last questionnaire (iMCQ) and therefore had missing data only for this questionnaire.

Drop-out analyses showed selective drop-out in the follow-up study compared to the original TraumaTIPS cohort (Mouthaan et al., 2014). The *n *= 194 participants in the follow-up study had significant higher education levels (*p* < .001), less often a non-Dutch origin (*p *< .001), were more often in a committed relationship (*p *= .007) compared to the *n *= 658 participants from the original cohort study who did not participate in the long-term follow-up at 1 year post-trauma. The *n *= 194 participants in the follow-up study also reported significant lower perceived impact of prior traumatic events at the initial assessment post trauma (*p *< .001), had significantly higher participation rates in the intermediate assessments of the original cohort study (*p*'s* *< .05) as well as lower PTSD symptoms (*p *= .016), and a lower prevalence of PTSD (*p *= .004) compared to the non-participants in the follow-up.

### 1.3. Measures

#### 1.3.1. CAPS-5 PTSD prevalence and symptom severity

The Clinician-Administered PTSD scale for DSM-5 (CAPS-5) was used to assess PTSD symptom severity and PTSD diagnosis over the last month (Blake et al., 1995; Weathers et al., 2018). The CAPS-5 is a standardized clinical diagnostic interview with 20 items on a 4-point Likert scale, ranging from 0 ‘absent’ to 4 ‘extremely’, each item corresponding to a specific DSM-5 symptom criterion for PTSD. Symptoms were assessed in relation to the index trauma of 12–15 years ago: participants were specifically instructed to answer keeping their index trauma in relation to their past research participation in mind. Following the CAPS-5 manual, we only counted symptoms with definite or probable connections as PTSD symptoms attributed to the index trauma of 12–15 years ago, minimizing the risk of overestimating the prevalence of long-term PTSD related to this traumatic event.

PTSD diagnosis was determined based on whether participants met the DSM-5 diagnostic criteria for PTSD, which required scoring 2 or higher on the following items: at least one intrusion item and avoidance item, two items on negative alterations in cognitions and mood, and two hyperarousal items (Weathers et al., 2018). Moreover, PTSD symptom severity was determined by summing the 20-item scores, resulting in a total score ranging from 0 to 80, with higher scores indicating higher PTSD symptom severity. We additionally determined subthreshold PTSD using the 4 most common definitions based on DSM-5 (see Supplementary file; Franklin et al., 2018), given the absence of a gold standard.

We used the Dutch-validated version of the CAPS-5 (Boeschoten et al., 2018). In the current study, we observed a high internal consistency of the CAPS-5 total score (*α* = .89). To ensure interrater reliability, every month a random selection of 15% of the audiotaped CAPS-5 interview was scored by a second rater not involved in the original follow-up clinical interview. We obtained excellent agreement on PTSD diagnosis (Cohen's kappa = 1) and PTSD symptom severity (ICC = .80).

We also measured the PTSD Checklist for DSM-5 (PCL-5) a self-report questionnaire for screening probable PTSD diagnosis and PTSD symptom severity (Blevins et al., 2015). Whilst we performed our subsequent analyses using the gold standard clinical interview (i.e. CAPS-5), it is of relevance to note that we observed high convergence between PCL-5 and CAPS-5 total scores in our sample (Hoeboer et al., 2024).

#### 1.3.2. Psychological outcomes

##### 1.3.2.1. HADS anxiety symptom severity

Anxiety symptom severity was measured using the anxiety subscale of the Dutch self-report Hospital anxiety and depression scale (HADS; Spinhoven et al., 1997). This subscale consists of 7 items with a 4-point Likert scale assessing anxiety symptom severity over the past week. Total scores were calculated by summing all items, resulting in a total score ranging from 0 to 28, with higher scores indicating higher symptom severity. The current study showed high internal consistency for the HADS anxiety subscale (*α* = .83).

##### 1.3.2.2. QIDS-SR depression symptom severity

Depression symptom severity was measured using the quick inventory of depressive symptomatology, self-rated version (QIDS-SR; Rush et al., 2003). The QIDS-SR consists of 16 items on a 4-point Likert scale assessing symptoms for major depressive disorder in the past week according to the DSM-IV. Total scores were calculated by summing all items, resulting in a total score ranging from 0 to 27, with higher scores indicating higher symptom severity. The current study showed good internal consistency of the QIDS-SR total score (*α* = .79).

##### 1.3.2.3. AUDIT alcohol use

Alcohol use over the past year was measured using the Alcohol Use Disorders Identification Test (AUDIT; Bush et al., 1998). The AUDIT includes 10 items on a 5-point Likert scale, ranging from 0 ‘never’, to 4 ‘(almost) daily’. Total scores were calculated by summing all items, resulting in a scale, ranging from 0 to 40, with higher scores indicating more alcohol use. The current study showed good internal consistency for the AUDIT total score (*α* = .78).

#### 1.3.3. Functional outcomes

##### 1.3.3.1. WHO-5 Well-being

Subjective psychological well-being was measured using the World Health Organization well-being index over the past two weeks (WHO-5; Topp et al., 2015). The WHO-5 includes 5 items on a 6-point Likert scale, ranging from 0 = ‘at no time’ to 5 = ‘all of the time’. Total scores were calculated by summing all items and multiplying by 4 to create a scale ranging from 0 to 100, with higher scores indicating higher well-being. The current study showed excellent internal consistency of the WHO-5 total score (*α* = .90).

##### 1.3.3.2. WHOQOL Quality of Life

Quality of life over the past two weeks was measured using the World Health Organization Quality of Life abbreviated (WHOQOL-BREF; Skevington et al., 2004). The WHOQOL-BREF includes 26 items on a 5-point Likert scale, ranging from 1 ‘worse outcome’ to 5 ‘best outcome’, measuring 4 domains on psychological, physical, social and environmental quality of life, and 2 items on general quality of life. Domain scores were converted to a total score ranging from 0 to 100, with higher scores indicating higher quality of life. The current study showed high internal consistency for psychological (*α* = .82), physical (*α* = .87), social (*α* = .72), and environmental (*α* = .81) quality of life.

##### 1.3.3.3. EQ-5D-5L Health-related Quality of Life

Health-related quality of life was measured using the EQ-5D-5L (European Quality of Life 5 dimensions 5 levels, Brooks & Group, 1996). The EQ-5D-5L includes 5 items on a 5-point Likert scale, ranging today's health-related symptoms from 0 = ‘no problems or symptoms’ to 4 = ‘not capable of or extreme symptoms’, measuring 5 dimensions: mobility, self-care, usual activities, pain/discomfort and anxiety/depression. We added an additional item measuring cognition (such as concentration and memory), with a similar item structure (Geraerds et al., 2019). The Dutch tariff for the EQ-5D-5L was used to calculate an index score, ranging from 0 (death) to 1 (full health), where a negative value is possible for health states worse than death (Dolan, 1997; Versteegh et al., 2016). The item on cognition was not included in the total score, but was used as an additional dimension, converted to a 0–100 scale. Moreover, the EQ-5D-5L also contained a Visual Analogue Scale (VAS) to measure overall health, where participants rate their health condition from 0 = ‘worst health imaginable’ to 100 = ‘best health imaginable’. The current study showed high internal consistency for the EQ-5D-5L index score (*α* = .82).

#### 1.3.4. Economic outcomes

##### 1.3.4.1. iMCQ Health Care Use

Health care use consumptions was measured using the Institute for Medical technology Assessment (iMTA) Medical Consumption Questionnaire (iMCQ; Bouwmans et al., 2015). The iMCQ is a non-disease specific questionnaire on health-care use, where participants are asked about their use of intramural medical care and extramural health care over the last 6 months. Intramural medical care consists of costs associated with an in-hospital stay, such as ambulance transportation to the ED, ED visit, medical specialist appointments, stay at a hospital ward, stay at intensive care unit (ICU), and diagnostics. Extramural health care consisted of medical costs outside the hospital, such as stay or day treatment in institutions such as nursing homes, rehabilitation centres or psychiatric institutions, homecare (domestic care, help with all day activities or nursing), visits to practitioners (general practitioner, company doctor, psychologist, social worker, physiotherapist, occupational therapist, speech therapist or dietician), and medication use. Health care costs were calculated by multiplying healthcare use per period with cost per unit in Euros for intramural and extramural care. The cost-reference manual was based on hospital price lists, previous research and the NZa (Dutch health care authority, 2017; Erasmus, 2016; Geraerds et al., 2019).

##### 1.3.4.2. iPCQ Productivity Loss

Productivity loss at work was only measured for participants who were employed during the follow-up assessment, using the iMTA Productivity Cost Questionnaire (iPCQ; Bouwmans et al., 2015). The iPCQ was used to assess how health-related issues affected productivity in the workplace over the last 6 months. Participants were asked to fill out questions on absenteeism (i.e. absence of work due to health-related issues) and presenteeism (i.e. being present at work but being less productive due to health-related issues). Productivity loss at work was calculated combining the costs associated with absenteeism using the friction costs method, assuming that productivity loss is limited to the time required to find replacement for absent employees. In accordance with Dutch guidelines, we used a friction period of 85 days, meaning that any productivity loss longer than the friction period was set to 85 (Hakkaart-van Roijen et al., 2015; Mohnen et al., 2019). The costs of productivity loss were determined by multiplying the total number of hours of work missed with the hourly wage rate. The cost-reference manual included the average wage rates per gender of the Dutch population (Dutch health care authority, 2017; Erasmus, 2016; Geraerds et al., 2019).

Given that the iPCQ productivity loss at work is only measured within participants who were employed at the follow-up assessment 12–15 years post-trauma, we also conducted Pearson correlation analyses to assess potential associations between employment status (yes/no) and PTSD symptom severity.

#### 1.3.5. LEC-5 trauma exposure since index trauma

Potential trauma exposure since index trauma was assessed using the Dutch version of the Life Events checklist for the DSM-5 (LEC-5), a component of the CAPS-5 (Blake et al., 1995; Boeschoten et al., 2018; Weathers et al., 2013). The LEC-5 includes 16 different categories of potential traumatic events that could be either directly experienced, witnessed, encountered during work or learned about them happening to a close friend or family member. Participants were specifically asked about events occurring since the index trauma of 12–15 years ago. A total score of trauma exposure since index trauma was calculated. We computed a cumulative trauma exposure score by summing the occurrences across all categories, resulting in a range between 0 and 64, based on a recent validation study which showed that the LEC total score incorporating all categories of exposure demonstrated highest reliability compared to the directly experienced only score and a weighted score across types of exposure (Weis et al., 2022).

#### 1.3.6. CBS checklist chronic disorders

Chronic disorders were assessed using the Statistics Netherlands (CBS) checklist of present chronic disorders including 14 diseases and an ‘other’ as option where people can indicate other chronic health problems (Statistics Netherlands, 2003). The CBS checklist evaluates the presence of chronic disorders affecting the individual or requiring ongoing treatment within the last 5 years.

The assessed disorders included: heart conditions; vascular issues; lung problems; stroke consequences; neurological disorders; kidney disease; diabetes; osteoporosis, dementia, psychiatric conditions, severe back problems, joint degeneration, joint inflammation and cancer. We added an additional option for chronic complaints due to COVID-19 infection. We categorized the presence or absence of chronic disorders dichotomously (Haagsma et al., 2011).

#### 1.3.7. Gender

Gender was assessed during our follow-up assessment. We asked participants whether they self-identified as men, women or otherwise.

### 1.4. Statistical analyses

The statistical analyses plan of the current study was pre-registered on the Open Science Framework prior to conducting the analyses (OSF; Karchoud et al., 2023). Statistical analyses were performed using IBM SPSS Statistics Version 28.0.

We applied bootstrapping to all linear regression analyses given that CAPS-5 total scores violated the assumptions of normality of errors and homoscedasticity (Chernick, 2011).

#### 1.4.1. Long-term PTSD prevalence

Descriptive statistics were used to determine the CAPS-5 PTSD diagnosis in the whole sample as well as men and women separately. We additionally included prevalence rates of subthreshold PTSD. We also performed a Fisher's exact test to examine differences in long-term PTSD prevalence between women and men.

#### 1.4.2. Psychological, functional and economic outcomes

We calculated Pearson correlations between all continuous variables. For interpretation purposes we also calculated mean differences in psychological, functional and economic outcomes, divided between participants meeting the DSM-5 criteria for PTSD and no PTSD, given that it was not possible to formally test these due to a limited number of participants that were classified with PTSD.

Subsequently, 16 separate linear regression analyses were conducted with CAPS-5 total score on psychological (HADS anxiety domain; QIDS-SR total score; AUDIT total score), functional (WHO-5 total score; WHOQOL psychological domain; WHOQOL physical domain; WHOQOL social domain; WHOQOL environmental domain; WHOQOL general item 1; WHOQOL general item 2; EQ-5D-5L index score; EQ-6D-5L cognition domain; EQ-5D-5L VAS) and economic (iMCQ intramural domain; iMCQ extramural domain; iPCQ total score) outcomes. An interaction effect between gender and CAPS-5 total score and main effect of gender were included to investigate gender as potential moderator. In case of an insignificant interaction effect, another analysis was conducted without the interaction term to interpret the main effects (Hayes, 2017). Additionally, LEC-5 total score and dichotomous presence of medical disorders based on the CBS checklist were included as covariates (men and women did not significantly differ on these covariates [all *p*'s > .05]). In these analyses, we applied 1000 bootstrapping samples with a 95% confidence interval. We corrected the false discovery rate (FDR) for multiple comparisons of each independent variable (CAPS-5 total score; CBS checklist; gender) in the *n *= 16 separate analyses with different outcome variables, by using the Benjamini-Hochberg correction (Benjamini & Hochberg, 1995). This resulted in the following alpha corrections: *α* = .038 for CAPS-5 total score; *α* = .019 for CBS checklist; *α* = .009 for gender. No alpha corrections were required for LEC-5 total score and the interaction effect of CAPS-5 total score and gender, as none of these multiple tests yielded significant results.

## 2. Results

### 2.1. Sample characteristics

**Figure 1.**
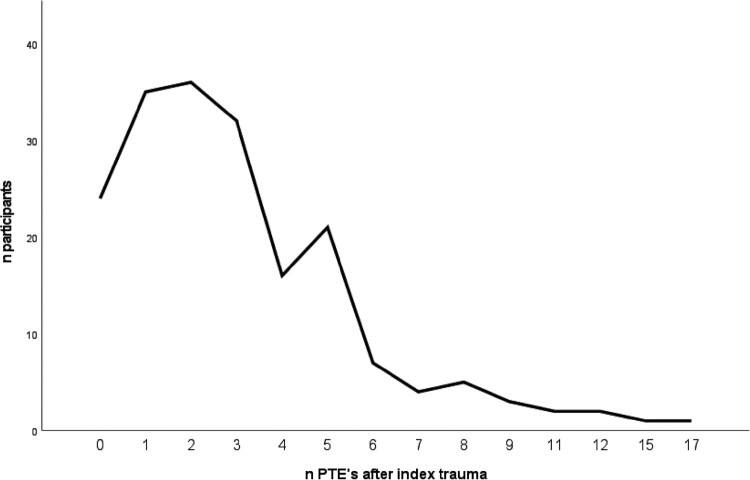
Number of potential traumatic events (PTE's) after index trauma according to the LEC-5. Note. LEC-5 = Life Events checklist for the DSM-5.

Most participants experienced at least one new potentially traumatic event after the index trauma (86.6%), with the number of new experienced events ranging from 0 to 17 (see Figure 1). Most commonly reported were life-threatening illness or injury (57.2%), severe human suffering (40.2%), transportation accident (26.8%) and sudden accidental death (22.2%) after their index trauma. Somewhat more than half of the participants suffered from a chronic disorder (56.2%). See Table 1 for all sample characteristics of all participants.

**Table 1.** Sample characteristics, divided between participants meeting the DSM-5 criteria for PTSD and not meeting the PTSD criteria.

	Total (*N *= 185) *n*	PTSD (*n = *9) *n*	No PTSD (*n* = 176) *n*
Gender (women)	65 (34.2%)	2 (22.2%)	63 (35.8%)
Age in years, *M* (*SD*)	54.47 (12.4)	50.44 (8.93)	54.49 (13.8%)
Netherlands as country of origin	170 (91.4%)	9 (100%)	161 (91.48%)
Relationship status			
Married/cohabitating/committed relationship	160 (84.2%)	5 (55.6%)	155 (88.1%)
Divorced/widowed	14 (7.4%)	1 (11.1%)	13 (7.4%)
No committed relationship	16 (8.4%)	3 (33.3%)	13 (7.4%)
Children (yes)	143 (75.3%)	5 (55.6%)	138 (78.4%)
Currently employed	124 (65.6%)	2 (22.2%)	122 (69.3%)
Education, highest completed			
Primary education/high school/secondary education	51 (26.9%)	2 (22.2%)	49 (27.8%)
Secondary vocational education	55 (28.9%)	6 (66.6%)	49 (27.8%)
Higher vocational education or University	84 (44.2%)	1 (11.1%)	83 (47.2%)
Index trauma, type			
Traffic accident	127 (67.2%)	5 (55.6%)	122 (69.3%)
Physical violence	4 (2.1%)	2 (22.2%)	2 (1.1%)
Work-related accident	25 (13.2%)	1 (11.1%)	24 (13.6%)
Fall from height	22 (11.6%)	0 (0%)	22 (12.5%)
Other	11 (5.8%)	1 (11.1%)	10 (5.7%)
Chronic disorders (yes)	109 (56.2%)	8 (88.9%)	101 (57.4%)

### 2.2. Long-term PTSD prevalence

See Table 2 for prevalence rates of long-term PTSD in the whole sample and per gender. The long-term prevalence of PTSD in the whole sample was 4.8%, with *n = *9 participants meeting the DSM-5 diagnostic criteria for PTSD in relation to the initial index trauma. Prevalence rates for subthreshold PTSD across the 4 commonly used definitions ranged between *n* = 0 (0%) and *n* = 7 (3.8%) participants (see Supplementary file).

**Table 2.** Prevalence rates of long-term PTSD according to DSM-5 diagnostic criteria in relation to the index trauma in the whole sample and per gender.

	PTSD	No PTSD
	*n*	*n*
Total	9 (4.8%)	176 (95.4%)
Female	2 (3.1%)	62 (96.9%)
Male	7 (5.8%)	114 (94.2%)

**Figure 2.**
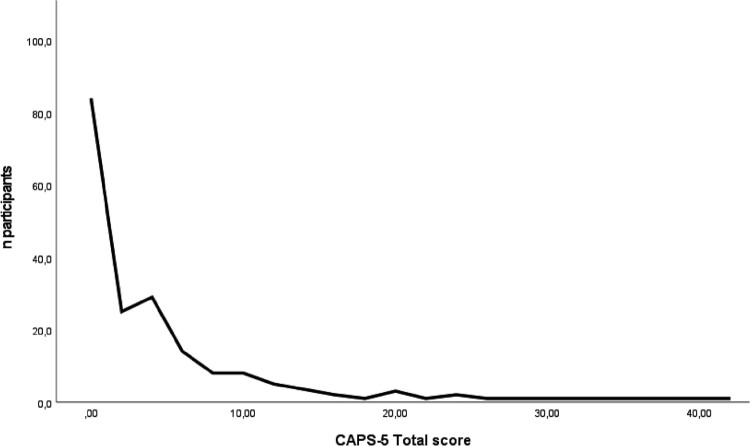
Distribution of CAPS-5 total scores. Note. CAPS-5 = The Clinician-Administered PTSD scale for DSM-5.

Fisher's exact test showed no significant gender differences in long-term PTSD prevalence, *p *= .721. There was also no significant gender difference in CAPS-5 PTSD symptom severity, *p *= .896. CAPS-5 total scores ranged between 0 and 41 (*M *= 4.08, *SD *= 7.08, see Figure 2). Participants meeting the DSM-5 criteria for PTSD had a mean PTSD symptom severity of 28.44 (*SD *= 7.42), ranging from 18 to 41. Participants without meeting the DSM-5 criteria for PTSD had a mean of 4.07 (*SD *= 7.08), ranging from 0 to 24.

See Table 1 for sample characteristics divided between participants meeting the DSM-5 criteria for PTSD and no PTSD.

### 2.3. Correlations between continuous variables

Table 3 displays all Pearson correlations between the continuous study variables.

**Table 3.** Pearson correlations between continuous study variables.

	CAPS-5 total	LEC-5 total	HADS anxiety	QIDS-SR total	AUDIT total	WHO-5 total	WHOQOL Psychological	WHOQOL Physical	WHOQOL Social	WHOQOL Environmental	WHOQOL General 1	WHOQOL General 2	EQ-5D-5L VAS	EQ-5D-5L cognition	EQ-6D-5L total	iPCQ total	iMCQ intramural	iMCQ extramural
CAPS-5 total	1.00	.21**	.51**	.64**	.12	−.47**	−.52**	−.52**	−.30**	−.31**	−.51**	−.33**	−.41**	−.30**	−.46**	−.00	.14	.21**
LEC-5 total		1.00	.09	.16*	.04	−.12	−.08	−.17*	−.12	−.11	−.18*	−.24**	−.13	.04	−.06	.10	.22**	.19*
HADS anxiety			1.00	.66**	.04	−.67**	−.68**	−.51**	−.38**	−.45**	−.49**	−.35**	−.44**	.37**	−.36**	−.06	.12	.15*
QIDS-SR total				1.00	−.02	−.64**	−.66**	−.67**	−.39**	−.44**	−.61**	−.45**	−.56**	.52**	−.55**	.04	.18*	.22**
AUDIT total					1.00	−.08	.02	.07	.05	.07	.04	.02	.02	−.07	.08	.02	.13	−.09
WHO-5 total						1.00	.78**	.69**	.57**	.53**	.63**	.54**	.58**	−.42**	.44**	.00	−.03	−.22**
WHOQOL Psychological							1.00	.71**	.69**	.68**	.68**	.46**	.51**	−.43**	.43**	−.03	−.03	−.21**
WHOQOL Physical								1.00	.56**	.60**	.61**	.61**	.63**	−.44**	.57**	−.03	−.21**	−.24**
WHOQOL Social									1.00	.56**	.57**	.35**	.38**	−.26**	.28**	−.07	.05	−.12
WHOQOL Environmental										1.00	.51**	.36**	.44**	−.32**	.42**	.03	.03	−.15*
WHOQOL General 1											1.00	.57**	.57**	−.39**	.47**	−.04	−.06	−.11
WHOQOL General 2												1.00	.55**	−.34**	.42**	−.03	−.24**	−.12
EQ-5D-5L total													1.00	−.40**	.57**	.09	−.05	−.14*
EQ-6D-5L Cognition														1.00	−.33**	−.12	.07	.08
EQ-5D-5L VAS															1.00	.07	−.14	−.14*
iPCQ total																1.00	.45**	.00
iMCQ intramural																	1.00	.19*
iMCQ extramural																		1.00

Note. CAPS-5 = The Clinician-Administered PTSD scale for DSM-5; LEC-5 = Life Events checklist for the DSM-5; HADS = Hospital anxiety and depression scale; QIDS-SR = quick inventory of depressive symptomatology, self-rated; AUDIT = Alcohol Use Disorders Identification Test; WHO-5 = World Health Organization well-being index; WHOQOL = World Health Organization Quality of Life abbreviated; EQ-5D-5L = European Quality of Life 5 dimensions 5 levels; iPCQ = Institute for Medical technology Assessment (iMTA) Productivity Cost Questionnaire; iMCQ = Institute for Medical technology Assessment (iMTA) Medical Consumption Questionnaire.

* = Correlation is significant at the *p*/alpha 0.05 level (two-tailed). ** = Correlation is significant at the 0.01 level (two-tailed).

### 2.4. Psychological, functional and economic outcomes

See Table 4 for an overview of mean differences in psychological, functional and economic outcomes between participants meeting and not meeting DSM-5 criteria for PTSD. Given the limited number of participants meeting DSM-5 criteria for PTSD, it was not possible to statistically test for potential differences in psychological, functional and economic outcomes.

**Table 4.** Mean differences in psychological, functional and economic outcomes, divided between participants meeting the DSM-5 criteria for PTSD and no PTSD.

	Total (*N *= 184) *M (SD)*	PTSD (*n *= 9) *M (SD)*	No PTSD (*n *= 176) *M (SD)*
Psychological			
HADS Anxiety	3.44 (3.21)	8.78 (2.39)	3.04 (2.77)
QIDS-SR Depression	4.13 (3.97)	11.67 (4.09)	3.67 (3.39)
AUDIT Alcohol use	4.15 (3.74)	7 (8.53)	3.9 (3.2)
Functional			
WHO-5 well-being	68.63 (19.49)	40 (16.73)	70.66 (17.88)
WHOQOL Psychological	73.93 (15.53)	48.15 (14.45)	75.62 (17.27)
WHOQOL Physical	74.04 (18.17)	49.21 (16.73)	75.62 (13.88)
WHOQOL Social	72.37 (17.93)	53.7 (21.29)	73.72 (16.89)
WHOQOL Environmental	82.45 (13.7)	67.01 (18.57)	83.56 (12.69)
WHOQOL General1	4.25 (.76)	2.89 (.93)	4.34 (.648)
WHOQOL General2	3.78 (1.01)	2.56 (.88)	3.85 (96)
EQ-5D-5L Index	0.96 (.09)	0.86 (.15)	0.96 (.08)
EQ-6D-5L Cognition	89.37 (16.29)	75.56 (21.86)	90 (15.86)
EQ-5D-5L VAS Overall health	79.03 (15.44)	62.56 (19.94)	80.25 (14.08)
Economic			
iPCQ Productivity loss	€1362.35 (3872.83)	€3095.94 (2915.83)	€1163.91 (3488.95)
iMCQ Intramural hospital costs	€414.59 (1567.21)	€916.44 (1629.30)	€384.37 (1582.67)
iMCQ Extramural hospital costs	€836.25 (2858.93)	€2378.10 (4354.03)	€778.01 (2796.37)

### 2.5. Cross-sectional associations between PTSD symptom severity and psychological outcomes

See Table 5 for the results of the linear regression analyses for psychological outcomes. Results showed that CAPS-5 PTSD symptom severity was significantly and positively associated with HADS anxiety (*β* = 0.53, *p *< .001) and QIDS-SR depression (*β* = 0.61; *p *< .001) upon Benjamini-Hochberg correction. CAPS-5 PTSD symptom severity was not significantly associated with AUDIT alcohol use (*p *= .223).

**Table 5.** Overview of separate regression analyses on psychological outcomes

	*B*	*SE* (B)	*β*	*p*	95% CI (*B*)
HADS anxiety					
CAPS-5 PTSD symptom severity	0.22	0.03	0.53	<.001*	0.17;0.27
LEC-5 Trauma exposure	−0.04	0.07	−0.03	.586	−0.17;0.10
CBS Chronic disorder	−0.33	−0.00	−0.06	.386	−1.08;0.38
Gender (reference = men)	1.21	0.41	0.19	.005*	0.38;2.01
QIDS-SR depression					
CAPS-5 PTSD symptom severity	0.33	0.04	0.61	<.001*	0.26;0.43
LEC-5 Trauma exposure	0.02	0.08	0.01	.827	−0.14;0.18
CBS Chronic disorder	0.71	0.45	0.09	.115	−0.18;1.57
Gender (reference = men)	0.49	0.45	0.06	.285	−0.42;1.37
AUDIT alcohol use					
CAPS-5 PTSD symptom severity	0.08	0.06	0.15	.223	−0.04;0.21
LEC-5 Trauma exposure	0.03	0.09	0.02	.728	−0.12;0.22
CBS Chronic disorder	−1.78	0.62	−0.16	.061	−2.45;−0.01
Gender (reference = men)	−1.94	0.47	−0.25	<.001*	2.88;−0.96

Note. CAPS-5 = The Clinician-Administered PTSD scale for DSM-5; LEC-5 = Life Events checklist for the DSM-5; CBS = Statistics Netherlands checklist; HADS = Hospital anxiety and depression scale; QIDS-SR = quick inventory of depressive symptomatology, self-rated; AUDIT = Alcohol Use Disorders Identification Test.

* = Significant effect based on Benjamini-Hochberg correction with an alpha correction of *α* = .038 for CAPS-5 total score; *α* = .019 for CBS checklist; *α* = .009 for gender. No alpha corrections were required for LEC-5 total score and the interaction effect of CAPS-5 total score and gender, as none of these multiple tests yielded significant results.

The overall model fit was significant for HADS anxiety, *F*(4, 180) = 19.29, *p* < .001, *R*^2^ = .30; QIDS-SR Depression, *F*(4, 180) = 32.43, *p* < .001, *R*^2^ = .42; and AUDIT Alcohol use, *F*(4, 180) = 5.24, *p* < .001, *R*^2^ = .10.

### 2.6. Cross-sectional associations between PTSD symptom severity and functional outcomes

See Table 6 for the results of the linear regression analyses for functional outcomes. Results showed that CAPS-5 PTSD symptom severity was significantly and negatively associated with all functional outcomes upon Benjamini-Hochberg correction: WHO-5 Well-being (*β* = −0.44, *p *< .001); WHOQOL Psychological (*β* = −0.52, *p *< .001); WHOQOL Physical (*β* = −0.44, *p *< .001); WHOQOL Social (*β* = −0.27, *p *= .002); WHOQOL Environmental (*β* = −0.28, *p *= .006); WHOQOL General 1 (*β* = −0.56, *p *< .001); WHOQOL General 2 (*β* = −0.23, *p *= .005); EQ-5D-5L Health-related QoL (*β* = −0.42, *p *< .001); EQ-6D-5L Cognition (*β*e = −0.29, *p *= .004); EQ-5D-5L VAS Overall health (*β* = −0.36, *p *< .001).

**Table 6.** Overview of separate regression analyses on functional outcomes.

	*B*	*SE* (B)	*β*	*p*	95% CI (B)
WHO-5 Well-being					
CAPS-5 PTSD symptom severity	−1.18	0.21	−0.44	<.001*	−1.63;−0.82
LEC-5 Trauma exposure	−0.06	0.43	−0.01	.898	−0.99;0.77
CBS Chronic disorder	−3.41	2.42	−0.09	.161	−8.32;1.58
Gender (reference = men)	−4.95	2.42	−0.12	.048	−0.88;−0.09
WHOQOL Psychological					
CAPS-5 PTSD symptom severity	−1.11	0.12	−0.52	<.001*	−1.34;−0.87
LEC-5 Trauma exposure	0.23	0.01	0.04	.463	−0.43;0.82
CBS Chronic disorder	−0.96	1.95	−0.03	.619	−4.95;3.11
Gender (reference = men)	−2.47	1.91	−0.08	.214	−6.40;1.25
WHOQOL Physical					
CAPS-5 PTSD symptom severity	−1.12	0.16	−0.44	<.001*	−1.47;−0.83
LEC-5 Trauma exposure	−0.27	0.31	−0.04	.377	−0.92;0.30
CBS Chronic disorder	−10.28	0.07	−0.28	<.001*	−14.65;−6.01
Gender (reference = men)	−6.22	2.18	−0.16	.006*	−10.69;−1.94
WHOQOL Social					
CAPS-5 PTSD symptom severity	−0.68	0.17	−0.27	.002*	−0.99;−0.32
LEC-5 Trauma exposure	−0.22	0.47	−0.04	.636	−1.25;0.67
CBS Chronic disorder	−3.62	2.64	−0.10	.174	−8.82;1.41
Gender (reference = men)	−0.14	2.55	−0.00	.953	−5.38;5.23
WHOQOL Environmental					
CAPS-5 PTSD symptom severity	−0.52	0.19	−0.28	.006*	−0.88;−0.14
LEC-5 Trauma exposure	−0.16	0.34	−0.03	.648	−0.80;0.54
CBS Chronic disorder	−2.93	1.90	−0.11	.108	−6.38;1.04
Gender (reference = men)	−1.47	2.02	−0.05	.474	−5.27;2.53
WHOQOL General 1					
CAPS-5 PTSD symptom severity	−0.05	0.01	−0.46	<.001*	−0.06;−0.03
LEC-5 Trauma exposure	−0.01	0.02	−0.05	.361	−0.04;0.02
Chronic disorder	−0.23	0.09	−0.16	.013*	−0.41;−0.05
Gender (reference = men)	0.05	0.10	0.04	.608	−0.15;0.24
WHOQOL General 2					
CAPS-5 PTSD symptom severity	−0.31	0.01	−0.23	.005*	−0.05;−0.01
LEC-5 Trauma exposure	−0.05	0.03	−0.15	.031	−0.11;−0.01
CBS Chronic disorder	−0.62	0.13	−0.31	<.001*	−0.87;−0.34
Gender (reference = men)	−0.08	0.14	−0.04	.586	−0.36;−0.17
EQ-5D-5L Health-related QoL					
CAPS-5 PTSD symptom severity	−0.01	0.00	−0.42	<.001*	−0.01;−0.00
LEC-5 Trauma exposure	0.00	0.00	0.07	.230	−0.00;0.01
CBS Chronic disorder	−0.04	0.01	−0.24	<.001*	−0.07;−0.02
Gender (reference = men)	−0.00	0.00	−0.01	.834	−0.02;0.02
EQ-6D-5L Cognition					
CAPS-5 PTSD symptom severity	−0.67	0.22	−0.29	.004*	−1.13;−0.27
LEC-5 Trauma exposure	0.12	0.40	0.02	.786	−0.76;0.82
CBS Chronic disorder	−2.29	2.22	−0.07	.308	−6.51;2.16
Gender (reference = men)	−5.44	2.52	−0.16	.038	−10.36;−0.34
EQ-5D-5L VAS Overall health					
CAPS-5 PTSD symptom severity	−0.75	0.21	−0.36	<.001*	−1.14;−0.32
LEC-5 Trauma exposure	−0.09	0.38	−0.02	.785	−0.85;0.70
CBS Chronic disorder	−6.35	1.83	−0.21	.003*	−9.99;−2.75
Gender (reference = men)	−4.23	2.09	−0.14	.051	−8.38;−0.15

Note. CAPS-5 = The Clinician-Administered PTSD scale for DSM-5; LEC-5 = Life Events checklist for the DSM-5; CBS = Statistics Netherlands checklist; WHO-5 = World Health Organization well-being index; WHOQOL = World Health Organization Quality of Life abbreviated; EQ-5D-5L = European Quality of Life 5 dimensions 5 levels.

* = Significant effect based on Benjamini-Hochberg correction with an alpha correction of *α* = .038 for CAPS-5 total score; *α* = .019 for CBS checklist; *α* = .009 for gender. No alpha corrections were required for LEC-5 total score and the interaction effect of CAPS-5 total score and gender, as none of these multiple tests yielded significant results.

The overall model fit was significant for WHO-5 well-being, *F*(4, 180) = 14.28, *p* < .001, *R*^2^= .24; WHOQOL Physical, *F*(4, 180) = 26.81, *p* < .001, *R*^2^ = .37; WHOQOL Psychological, *F*(4, 180) = 17.57, *p* < .001, *R*^2^ = .28; WHOQOL Social, *F*(4, 180) = 5.24, *p* < .001, *R*^2^ = .10; WHOQOL Environmental, *F* = (4, 180) = 5.62, *p* < .001, *R*^2^ = .11; WHOQOL General 1, *F* = (4, 180) = 17.91, *p* < .001, *R*^2^ = .29; WHOQOL General 2, *F* = (4, 180) = 13.14, *p* < .001, *R*^2^ = .23; EQ-5D-5L Health-related quality of life, *F*(4, 180) = 16.40, *p* < .001, *R*^2^ = .27; EQ-6D-5L cognition, *F*(4, 180) = 6.25, *p* < .001, *R*^2^ = .12; and EQ-5D-5L VAS overall health, *F*(4, 180) = 13.84, *p* < .001, *R*^2^ = .24.

### 2.7. Cross-sectional associations between PTSD symptom severity and economic outcomes

In total *n = *124 participants (65.6%) were employed at follow-up, of which *n = *19 (7.3%) participants reported being present at work but being less productive (i.e. presenteeism) in the last 6 months, resulting in mean productivity costs of €674.55 for these participants with presenteeism. *N = *43 (36.3%) participants were (at least partially) absent of work due to health-related issues (i.e. absenteeism) in the last 6 months, including *n = *3 (2.4%) participants with long-term absenteeism (i.e. 97, 543 and 607 days). Mean productivity costs per participant with absenteeism were €3865.27. Results showed significant associations between CAPS-5 PTSD symptom severity and employment status in all participants (*r* = −0.24, *p *< .001) and in all participants within the current employment age in the Netherlands (18–66 years; *r* = −0.46, *p *< .001).

With respect to health care use, the highest intramural hospital costs included hospital treatment (ranging from €0 to €8460, *M *= €153.68, *SD *= €826.49), stay at hospital ward (ranging from €0 to €6795, *M *= €107.86, *SD *= €710.35), and medical specialist appointments (ranging from €0 to €4264, *M *= €107.16, *SD *= €350.88). The highest extramural hospital costs included medication costs (ranging from €0 to €21533.40, *M *= €470.84.16, *SD *= €2399.85), ward in a psychiatric institution (ranging from €0 to €9270, *M *= €49.05, *SD *= €674.29), and treatment in a psychiatric institution (ranging from €0 to €7500, *M *= €40.64, *SD *= €548.47).

See Table 7 for the results of the linear regression analyses for economic outcomes. CAPS-5 PTSD symptom severity was not significantly associated with iPCQ productivity loss (*p *= .742) and iMCQ extramural hospital costs (*p *= .130) and intramural hospital costs (*p *= .130).

**Table 7.** Overview of separate regression analyses on economic outcomes.

	*B*	*SE* (B)	*β*	*p*	95% CI (B)
iPCQ Productivity loss					
CAPS-5 PTSD symptom severity	−19.93	61.90	−0.03	.742	−161.32;93.49
LEC-5 Trauma exposure	124.70	145.49	0.10	.396	−112.41;457.92
CBS Chronic disorder	491.88	670.79	0.07	.510	−722.52;1894.25
Gender (reference = men)	386.96	650.72	0.05	.569	−839.16;1824.37
iMCQ Intramural hospital costs					
CAPS-5 PTSD symptom severity	14.30	17.30	0.06	.394	−19.55;53.27
LEC-5 Trauma exposure	108.68	80.48	0.19	.222	3.43;219.27
CBS Chronic disorder	478.02	194.98	0.15	.093	162.45;941.82
Gender (reference = men)	−106.14	−1.48	−0.03	.622	−532.06;261.86
iMCQ Extramural hospital costs					
CAPS-5 PTSD symptom severity	57.47	34.87	0.14	.130	0.00;136.94
LEC-5 Trauma exposure	156.26	93.67	0.15	.103	−22.35;352.21
CBS Chronic disorder	950.40	310.63	0.16	.015*	430.85;1652.29
Gender (reference = men)	903.86	473.99	0.15	.077	16.05;1886.51

Note. CAPS-5 = The Clinician-Administered PTSD scale for DSM-5; LEC-5 = Life Events checklist for the DSM-5; CBS = Statistics Netherlands checklist; iPCQ = Institute for Medical technology Assessment (iMTA) Productivity Cost Questionnaire; iMCQ = Institute for Medical technology Assessment (iMTA) Medical Consumption Questionnaire. * = Significant effect based on Benjamini-Hochberg correction with an alpha correction of *α* = .038 for CAPS-5 total score; *α* = .019 for CBS checklist; *α* = .009 for gender. No alpha corrections were required for LEC-5 total score and the interaction effect of CAPS-5 total score and gender, as none of these multiple tests yielded significant results.

The overall model fit was not significant for iPCQ productivity loss (*p* = .735). The overall model fit was significant for iMCQ intramural hospital costs *F*(4, 179) = 3.92, *p* = .004, *R*^2^ = .08, and for iMCQ extramural hospital costs *F*(4, 180) = 5.98, *p* < .001, *R*^2^ = .12. There was *n* = 1 missing on the iMCQ intramural hospital costs.

### 2.8. Gender effects

Men reported significantly higher HADS anxiety (*β* = 0.19, *p *= .005), lower AUDIT alcohol use (*β* = −0.25, *p < *.001), and lower WHOQOL physical-related quality of life (*p* = .006, *q* = .009) scores compared to women. Gender was not significantly associated with the other psychological, functional and economic outcomes, see Tables 5–7. There were no significant interaction effects of gender and CAPS-5 PTSD symptom severity on any psychological, functional and economic outcome.

## 3. Discussion

We examined the long-term prevalence of PTSD and associated adverse psychological, functional and economic outcomes 12–15 years following traumatic injury in adults. Almost 5% of participants met the DSM-5 diagnostic criteria for PTSD related to the incident of 12–15 years ago during which these injuries were inflicted. Furthermore, long-term PTSD symptoms were associated with more adverse psychological and functional outcomes. No significant gender differences in the long-term PTSD prevalence nor in its related psychological, function and economic outcomes were observed.

### 3.1. PTSD prevalence

Our study contributes to the existing literature as the first longitudinal study investigating PTSD prevalence more than a decade after individual civilian trauma. The observed long-term (full) PTSD prevalence rate of 4.8% and subthreshold PTSD prevalence rate of 0–3.8% at 12–15 years post-trauma likely represents a conservative estimate. This is because, firstly, we maintained a strict approach in attributing PTSD symptoms to the index trauma, to minimize the risk of overestimating the prevalence of long-term PTSD related to the index trauma. Furthermore, selective drop-out for this follow-up assessment compared to the previous TraumaTIPS cohort assessments (Mouthaan et al., 2014) resulted in significantly lower PTSD symptoms and prevalence rates at 1-year post-trauma in the current participants compared to those who did not participate. This could contribute to a lower prevalence rate in our follow-up.

Our observed PTSD prevalence seems relatively low compared to other studies with similar durations of follow-up in samples with uniformed services or collective trauma. For instance, PTSD prevalence rates were 10.5% in US veterans at 14 years after the Vietnam war (Koenen et al., 2003), 26.1% at 14 years after the genocide in Rwanda (Munyandamutsa et al., 2012), 16.7% at 12 years after the Hurricane Katrina in the US (Raker et al., 2019), 11% at 10–11 years after the 9/11 WTC attacks in US/New York police responders (Cone et al., 2015), and 13% at 14–15 years after 9/11 in WTC tower survivors (Adams et al., 2019). One potential factor of influence is that the conditional risk for PTSD is greatly influenced by the type of trauma experienced, and that motorized vehicle accidents and accidental injury as experienced by the majority of our sample is associated with relatively lower conditional PTSD risk (Liu et al., 2017). Furthermore, the current sample predominantly consisted of middle-aged adults living in the Netherlands. Although there was variation in education level and other socioeconomic status (SES) indicators; and a minority indicated a non-western origin, these participants were all currently living in a society characterized as WEIRD (Western, educated, industrialized, rich and democratic; Henrich et al., 2010). Aligning with data of the International Consortium to Predict PTSD (ICPP), including our cohort and 13 other studies on traumatic injury (Qi et al., 2018), lower prevalence rates of PTSD following traumatic injury are generally observed in WEIRD populations or samples with comparable socioeconomic status. This may be due to for example high-quality healthcare and potentially lower trauma severity. Nevertheless, their pooled analyses confirmed that these international cohort studies represent different samples from a common parent population of acute care trauma admissions (Shalev et al., 2019). Thus, while we cannot rule out the potential impact of the above mentioned factors on PTSD prevalence, the findings are likely still generalizable beyond our cohort.

All in all, our study contributes to the existing literature by supporting the widely recognized findings from epidemiological studies that adults affected by individual civilian trauma may develop long-term PTSD (Kessler et al., 2017). These potential long-term consequences highlight the importance of accurate screening and prevention for PTSD. Future research should investigate how short- to midterm outcomes relate to long-term outcomes. Furthermore, it would be relevant to examine whether previously identified risk factors of short-to-mid-term PTSD are associated with long-term PTSD.

### 3.2. Psychological, functional and economic outcomes

Across the whole sample of participants with and without long-term PTSD, we observed that higher PTSD symptom severity was associated with more adverse psychological outcomes i.e. higher anxiety and depressive symptoms. While this has already been established through extensive epidemiological studies assessing psychiatric diagnostic status (Spinhoven et al., 2014), our longitudinal study confirms this in a broader clinical population representing the entire range of PTSD symptom severity. In contrast to a 2 year follow-up study, we found no significant relationship between PTSD symptoms and alcohol use (Nickerson et al., 2014).

Higher PTSD symptom severity was associated with more adverse functional outcomes, i.e. reduced well-being and quality of life. This adds to existing research showing lower (health-related) quality of life and well-being several years after traumatic injury (Holtslag et al., 2007; Kellezi et al., 2017; Soberg et al., 2015; Vles et al., 2005). In addition, we observed that higher PTSD symptom severity was associated with reduced quality of life on a broad range of domains, including psychological, physical, social, environmental, general, health-related, cognition, overall health and well-being.

In contrast, higher PTSD symptom severity was not significantly associated with productivity and healthcare costs 12–15 years post-trauma. Our findings may partially be explained by the fact that productivity loss was only examined over the past 6 months and only in the two-thirds of our sample that was currently employed. In line with previous research (Davis et al., 2022) we observed a significant association between participants’ employment status and long-term PTSD symptom severity, indicating enduring productivity loss. Therefore we cannot rule out that higher PTSD symptom severity is associated with higher productivity costs over time. Zooming in on the descriptive statistics we did notice higher productivity and health care costs for participants meeting the diagnostic criteria for PTSD compared to those without PTSD. This is in line with previous research showing higher health-care costs in people with PTSD up to 5 years after trauma (Bothe et al., 2020). As unfortunately our sample size did not allow for testing this difference statistically, this should be further investigated in future research.

Thus, higher PTSD symptom severity was significantly associated with more adverse psychological and functional outcomes. Further research on the causality within these long-term interrelationships is needed to understand whether PTSD symptoms precede these adverse outcomes, or vice versa.

### 3.3. Gender differences

In the current study we did not observe gender differences in the long-term prevalence of PTSD. At first, these findings might seem counterintuitive given that meta-analytic research indicates higher conditional PTSD risk in women compared to men, even in the context of similar trauma exposure (Tolin & Foa, 2008). However, while our study specifically examined the PTSD prevalence related to a defined index traumatic event 12–15 years ago, in this previous meta-analysis the PTSD symptoms were specifically attributed to a specific index traumatic event in fewer than 10% of included studies. It would be interesting for future research to examine whether the reference event for PTSD assessment influences the magnitude of a gender effect, for example, by comparing different ways of PTSD assessment within a sample, similar to a study by Liu et al. (2017) which investigated associations of randomly selected traumatic events with DSM-IV Diagnosed PTSD. Nonetheless, our findings align with a recent systematic review and meta-analysis on longitudinal studies showing that while higher PTSD prevalence was present in women during more short-term post-trauma follow-up assessments, this difference was not present at assessments between 2 and 5 years post-trauma (Haering et al., 2022). However, whereas this meta-analysis showed that women had consistently higher PTSD symptom severity until 5 years post-trauma (Haering et al., 2022), this was not supported by our study showing no gender differences in long-term PTSD symptom severity. Collectively, ours and previous findings suggest that women may more frequently develop PTSD or high PTSD symptoms relatively early post-trauma, but also show more recovery over time (e.g. Diamond et al., 2022); while men may more frequently meet the PTSD diagnostic criteria at a later moment post-trauma (e.g. Berntsen et al., 2012; Boasso et al., 2015; Dickstein et al., 2010; Eekhout et al., 2016). This also aligns with previous findings in our cohort showing that women more often experienced high initial PTSD symptoms which recovered within the first year post-trauma, whereas men more often experienced low initial PTSD symptoms with a delayed onset of PTSD symptoms within the first year post-trauma (van Zuiden et al., 2022). Future research should investigate the mechanisms influencing these differences in PTSD course between men and women, including both sex-related factors such as reproductive phase (Michopoulos et al., 2023; Stevens et al., 2018) and gender-related factors such as masculine gender role stress (Christiansen & Berke, 2020). Yet, we should be careful when interpreting these findings, as the absence of gender differences could also be related to our imbalanced sample containing more men than women. Moreover, men in our sample experienced more interpersonal trauma, had lower education levels and higher injury severity than women (van Zuiden et al., 2022), which is typically associated with higher PTSD symptom severity and prevalence.

This was the first longitudinal study examining gender effects in these long-term interrelationships. We did not find significant gender differences in the correlations between long-term PTSD symptom severity and adverse psychological, functional and economic outcomes. Thus, gender did not moderate the relationship of PTSD symptom severity and adverse outcomes in our study 12–15 years post-trauma.

### 3.4. Strengths and limitations

This study has several limitations that warrant consideration. Within our long-term follow-up assessment we were unfortunately unable to establish contact with approximately one-third of the potential participants from the original cohort and whose contact details therefore likely were not correct anymore. This resulted in a rather modest sample size. This limited the statistical power to conduct statistical analyses on differences in our psychological, functional and economic outcomes of interest between adults meeting and not meeting the diagnostic criteria for PTSD. Nevertheless, we conducted regression analyses, allowing us to investigate cross-sectional associations between adverse psychological, functional and economic outcomes and the entire observed range of PTSD symptom severity. Additionally, there was selective drop-out when comparing the current participants to the complete original TraumaTIPS cohort (Mouthaan et al., 2014). Adults who participated in the follow-up study had significant lower PTSD symptoms at 1 year post-trauma and lower prevalence of PTSD at 1 year follow-up (probably contributing to the relatively low prevalence rate in our follow-up), subjectively less impactful previous potential traumatic events, higher education levels, more often a relationship and higher participation rates in the intermediate assessments compared to those who did not participate. Moreover, our exclusion criteria regarding the presence of moderate to severe traumatic brain injury related to the index trauma at inclusion and regarding the presence of severe neurological conditions clearly associated with impaired cognition at follow-up, could have also excluded those adults at high risk for PTSD (Bryant et al., 2015). These inclusion biases may limit the representativeness of our sample and we should take precaution in generalizing our findings.

Furthermore, it is crucial to acknowledge that our study design is strictly observational and that investigated correlations were cross-sectional, meaning that we cannot draw any causal inferences from our findings. Although we corrected for false positive rates within our analyses using the Benjamini-Hochberg correction (Benjamini & Hochberg, 1995), we also acknowledge the possibility that observed associations could be indirectly influenced by other outcome variables incorporated in the current study. Moreover, given that each participant is reporting on all our measures this could result in a shared reporting bias, also potentially increasing associations between constructs.

Notably, within our regression analyses we controlled for the influence of existing chronic disorders and additional trauma exposure since the index trauma. However, although we assessed PTSD symptoms specifically in relation to the index trauma of 12–15 years ago, we cannot exclude the possibility that later trauma exposure affected these symptoms*.* Moreover, we did not examine effects of potential previous PTSD treatment on the long-term PTSD prevalence and associated adverse outcomes.

## 4. Conclusion

Overall, our findings underscore the long-term presence of PTSD in a proportion of adults who experienced (suspected) serious traumatic injuries more than a decade ago. That is, 12–15 years after a (suspected) serious traumatic injury almost 5% met the diagnostic criteria for PTSD related to the event during which these injuries were inflicted. The long-term PTSD symptom severity was associated with higher anxiety and depression symptoms, lower well-being and quality of life. PTSD is already widely recognized for its substantial impact in the aftermath of a trauma. The current study emphasis the potential long-term consequences of traumatic events such as traffic accidents or physical assault, highlighting the importance of accurate screening and prevention for PTSD concerning individual civilian trauma.

## Supplementary Material

Supplementary_file_EJPT.docx

## Data Availability

The data of the study and code to produce the results described in this paper are available at Open Science Framework (OSF; https://osf.io/82rdt). The study will be registered in the FAIR Traumatic Stress Data Sets library of the Global Collaboration on Traumatic Stress (GCTS).
