# Supplementary material for: Long-term PTSD prevalence and associated adverse psychological, functional, and economic outcomes: a 12–15 year follow-up of adults with suspected serious injury
Source: Eur J Psychotraumatol. 2024 Sep 19;15(1):2401285. doi: 10.1080/20008066.2024.2401285 (PMC11414644; doi:10.1080/20008066.2024.2401285)
Supplement: Supplementary_file_EJPT.docx [file ZEPT_A_2401285_SM3234.docx]

**Supplementary file**

**Table 1**

*Most Common Subthreshold PTSD definitions (Franklin et al., 2018)*

| Definition | Criteria based on DSM-5 |
| --- | --- |
| Definition-1 | Option A: Meeting criteria for intrusion and avoidance, plus changes in cognitions and mood Option B: Meeting criteria for intrusion, changes in cognitions and mood, plus arousal and reactivity |
| Five-symptom | Meeting at least 1 symptom in each symptom cluster, but not meeting criteria for full-threshold PTSD |
| Majority | Meeting 3 of the 4 symptom clusters |
| Six-plus | Having at least 6 symptoms of PTSD irrespective of which cluster, but not meeting full-threshold criteria due |

**Table 2**

Prevalence Rates of subthreshold PTSD According to DSM-5 Diagnostic Criteria (4 most common definitions) in Relation to the Index Trauma within the whole sample and per Gender.

|  | CAPS-5 Subthreshold PTSD | |  | |
| --- | --- | --- | --- | --- |
|  | Definition-1 | 5-symptom | Majority | Six-plus |
|  | n | n | n | n |
| Total | 0 | 7 | 2 | 5 |
| Female | 0 | 2 | 2 | 2 |
| Male | 0 | 5 | 0 | 3 |
